# Dietary Protein, Exercise, and Frailty Domains

**DOI:** 10.3390/nu11102399

**Published:** 2019-10-08

**Authors:** Josje D. Schoufour, Elvera Overdevest, Peter J. M. Weijs, Michael Tieland

**Affiliations:** 1 Faculty of Sports and Nutrition, Center of Expertise Urban Vitality, Amsterdam University of Applied Sciences, 1097 DZ Amsterdam, The Netherlands; j.d.schoufour@hva.nl (J.D.S.); e.overdevest@hva.nl (E.O.); p.j.m.weijs@hva.nl (P.J.M.W.); 2 Faculty Health, Center of Expertise Urban Vitality, Amsterdam University of Applied Sciences, 1097 DZ Amsterdam, The Netherlands; 3 Amsterdam University Medical Centers, University of Amsterdam, 1012 WX Amsterdam, The Netherlands

**Keywords:** frailty, protein, physical activity

## Abstract

Increasing awareness of the impact of frailty on elderly people resulted in research focusing on factors that contribute to the development and persistence of frailty including nutrition and physical activity. Most effort so far has been spent on understanding the association between protein intake and the physical domain of frailty. Far less is known for other domains of frailty: cognition, mood, social health and comorbidity. Therefore, in the present narrative review, we elaborate on the evidence currently known on the association between protein and exercise as well as the broader concept of frailty. Most, but not all, identified studies concluded that low protein intake is associated with a higher prevalence and incidence of physical frailty. Far less is known on the broader concept of frailty. The few studies that do look into this association find a clear beneficial effect of physical activity but no conclusions regarding protein intake can be made yet. Similar, for other important aspects of frailty including mood, cognition, and comorbidity, the number of studies are limited and results are inconclusive. Future studies need to focus on the relation between dietary protein and the broader concept of frailty and should also consider the protein source, amount and timing.

## 1. Introduction

Frailty is a complex cascade that involves several age-related physiological alterations eventually leading to loss of function and failure to respond to stress [1]. Frail people are in a state of increased vulnerability to adverse health outcomes including diseases, hospitalization and early death [1].

Although experts generally agree upon the concept of frailty, there is an ongoing debate on how to operationalize and measure frailty in elderly people. There are two major frailty operationalizations, the physical frailty phenotype, and the multidimensional frailty phenotype. The first phenotype, originally operated by Fried and colleagues, is based on five core clinical features that together form the frailty phenotype: unintentional weight loss, low muscle strength, feeling of exhaustion, reduced physical activity capacity and slow walking speed [2]. The second phenotype, often operationalized as the frailty index [3,4], includes physical impairments, mood, cognition, social environment, comorbidity and disabilities [5]. Where the physical frailty phenotype is based on pre-defined physical variables, the multidimensional frailty focuses on the total number of deficits in an individual and pays less attention to the exact nature of the problems. Both measures have been applied in several populations and have shown predictive validity in terms of survival and health indicators [5,6,7,8].

As our society is ageing, frailty will become a major threat. As a result, research has focused into factors that contribute to the development and persistence of frailty. Nutrition and physical activity are two factors closely related to frailty. Inadequate energy, macronutrient and/or micronutrient intake are associated with both the physical frailty phenotype and the multidimensional frailty phenotype [9,10]. Most efforts so far have been spent on understanding the association and relation between protein intake and the physical frailty phenotype [11]. Dietary protein plays an essential role in maintaining skeletal muscle mass in elderly people. Low dietary protein intake, especially when accompanied with low physical activity, has been linked to progressive loss of muscle mass leading to loss of muscle function and low muscle strength, low walking speed, and feeling of exhaustion, all associated with the physical frailty phenotype [12,13,14,15]. Indeed, several randomized controlled trials observed that the combination of physical activity and protein intake have a beneficial effect on muscle mass and muscle function and can help to prevent frailty [16,17]. Far less is known on the association between protein intake with or without physical activity and the broader concept of frailty (including the frailty index). On one hand, dietary protein deficiency may impact several physiological systems, including the immune system [18] and bone health [19,20]. On the other hand, excessive protein use may negatively influence health outcomes [21,22]. Therefore, in the present narrative review, we elaborate on the evidence currently known on the association between protein and physical activity and the broader concept of frailty.

## 2. Dietary Protein Intake and Recommendations in Frail Older Adults

Dietary protein intakes have been recorded among multiple studies and show similar observations for frail older people. Among others, Tieland et al. showed a dietary protein intake of 1.0 g/kg/d in community-dwelling frail older people [23], but lower intakes have been found for institutionalized frail older adults (0.8 g/kg/d). Although these intakes are above the recommended dietary allowance of 0.8 g/kg/d, 10% of the community-dwelling elderly people and frail elderly people, and 35% of the institutionalized frail elderly people fail to meet the minimum protein intake required to sustain overall health [23]. Moreover, to maintain muscle mass and physical performance even higher protein intakes up to 1.0–1.2 g/kg/d or even 1.2–1.5 g/kg/d have been suggested [23,24,25,26]. Considering these recommendations, more than 90% of the frail population might not meet these recommendations and higher protein intake in frail elderly people are suggested to attenuate the onset of frailty.

## 3. Dietary Protein Intake and Frailty Domains

The broader multidimensional frailty phenotype considers several domains, including physical health, disability, mood, cognition, social wellbeing, and comorbidity, that together determine the frailty status. The most well-known and applied operationalization of this approach is the frailty index, based on the accumulation of deficits from a broad range of health domains [4]. Although the frailty index has been applied extensively to study aging and trajectories of aging, the association with protein intake and/or physical activity is less well considered. In general, the few studies that do look into the association between physical activity and the frailty index observe less frailty with increasing levels of physical activity [27,28,29,30]. Similar, approaches that looked into multidimensional constructs other than the frailty index also observed a positive effect of physical activity [31]. To our knowledge only one study looked into the association between a combination of frailty domains and protein intake, using the frailty index. They observed that a high protein intake was associated with a higher frailty index (worse frailty status) over time [32] when adjusting for total energy intake. This association was completely driven by animal protein. It could be that this association reflects the association between poor dietary quality (high in animal products) and higher levels of frailty. This hypothesis is in line with previous findings that show that adherence to a diet high in animal products or low adherence to dietary guidelines that stimulate a plant-based diet are associated with higher frailty levels [7,10,30,33]. Additionally, a recent cohort study shows that adhering to a higher quality diet, high in plant protein, lead to an attenuation of frailty in older adults [34]. Although very few studies investigate the association between the multidimensional frailty phenotype and protein intake, several efforts were made to understand the association between protein intake and several important aspects of frailty. Below we elaborate on the effects of dietary protein intake on different frailty domains: physical frailty (including physical disability), mood, cognition, and comorbidity. 

### 3.1. Protein Intake and Physical Frailty

The physical frailty phenotype is operationalized as a biological syndrome, based on a cluster of symptoms that are commonly observed in frail older people: unintended weight loss, low physical activity, weakness, slowness and fatigue [2]. It is hypothesized that inadequate dietary protein may be associated with physical frailty and that increasing dietary protein intake may attenuate the onset of physical frailty or even improve the cluster of physical frailty. Indeed, a recent meta-analysis showed that inadequate protein intake is associated with higher frailty prevalence’s in older adults [15]. In this analysis of observational studies, the heterogeneity was high, indicating large inconsistencies among studies. Whereas the majority showed that adequate dietary protein intake is associated with lower risk for or a lower prevalence of physical frailty [12,13,14,35], some did not observe an association between protein intake and physical frailty [36,37]. The apparent contradictory findings may be due to the adaptations made to the Fried criteria and cut-off points in most of these studies and differences in analytical techniques (e.g., important confounders and effect modifiers including total energy intake, physical activity and BMI). For example, associations between dietary protein intake and frailty can be confounded by energy intake in which a positive energy balance, rather than protein intake was associated with frailty prevalence [37]. Despite these differences in results, pooled analysis showed that inadequate protein intake is associated with a higher frailty prevalence in older adults [15]. 

Multiple human intervention studies have been performed to study the impact of increased dietary protein intake on muscle mass, physical performance, and frailty. In line with the observational studies, there are some discrepancies between studies. Whereas some studies show a beneficial effect of dietary protein on muscle mass [16] or physical performance [17] in frail elderly people, other studies did not find an effect of increased dietary protein intake on muscle mass, strength or performance in elderly people that consume adequate daily protein [38,39]. An explanation for these apparent differences between studies include differences in design, outcomes measures, dietary protein strategy, and the inclusion of exercise. 

### 3.2. Protein Intake and Mood

The observed association between dietary protein intake and mood is mainly found as an indirect effect of dietary protein on maintaining weight or weight loss. A high protein intake in a low energy diet can help to maintain or lose weight. In obese or overweight participants, it has been observed that weight loss is frequently, but not always, associated with a higher quality of life [40,41,42]. Nevertheless, in a recent RCT among adults with obesity and type 2 diabetes, weight loss itself was associated with an increase in quality of life, but it did not matter whether this was achieved with a high or low protein diet [41]. There are a few observational studies that evaluated the association between protein intake and components of mood including mental wellbeing, depression, and anxiety. Generally, these studies do not find an association between protein intake and mood [43,44]. In conclusion, at this moment there is very little evidence supporting a direct link between protein intake and mood. 

### 3.3. Protein Intake and Cognition

The role of protein intake on cognitive performance is not well understood and only a few studies report on the association between protein intake and cognition. Observational studies found that high protein intake was associated with better cognitive performance [45,46,47]. Nevertheless, such associations are prone to confounding and reversed causation. Trials investigating the short time effect of protein intake on cognitive performance generally observed small improvement [48,49,50], but not all trials observe this effect [51]. An RCT investigating the effect of protein supplementation on long term effects (24 weeks) in prefrail and frail elderly found that the protein group (1.4 g protein/kg/d) significantly improved reaction time (executive functioning), but not in other cognitive functions compared to the placebo group (1.0 g protein/kg/d) [52]. In another RCT among pilot’s there was no effect of protein intake on memory [53]. At this moment there is limited evidence that cognitive performance is influenced by dietary protein intake [54,55]. Trials with longer follow-up among elderly people are needed in order to determine whether protein, in general or specific amino acids, may reduce the occurrence of cognitive limitations.

### 3.4. Protein Intake and Comorbidity

It is beyond the scope of this paper to discuss all diseases and conditions and how protein intake may beneficially or harmfully influence incidence and severeness of the condition. We will briefly touch upon a few common diseases and conditions that have been frequently associated with dietary protein intake.

#### 3.4.1. Metabolic Disease

Dietary protein has been shown effective for body-weight management and related risk factors including adiposity, blood pressure, and triglyceride levels [56,57]. On the short-term, protein enhances satiety and increases energy expenditure caused by a combination of factors including anorexigenic hormone concentrations and increased thermogenesis [58]. In the long-term, when losing weight sufficient protein intake results in better fat free mass preservation [59]. A high body weight, or more specifically a high fat mass, is associated with several non-communicable diseases including type 2 diabetes, cardiovascular diseases and non-alcoholic fatty liver disease (NAFLD). Following the generally positive effect of high-protein diets it is, therefore, hypothesized that it would also beneficially influence the prevention of several non-communicable diseases. Indeed, risk factors for cardiovascular and metabolic diseases generally change in a favorable direction during weight loss. Even so, if a high protein diet also beneficially influences risk factors beyond the effect of weight loss remains inconclusive [60,61,62]. For example, high protein diets applied during weight loss and weight maintenance show favorable effects on insulin sensitivity but it is unlikely that protein itself promotes insulin sensitivity beyond the effect of weight loss [60].

#### 3.4.2. Cardiovascular Disease

It is difficult to make clear conclusions regarding the direct relation between dietary protein intake and cardiometabolic health because overweight and obesity are established risk factors for negative cardiometabolic outcomes. In general, current evidence, mainly from cohort studies and short-term clinical trials, does not show a clear association between dietary protein intake and risk for coronary heart disease, stroke or blood pressure [61,63,64,65]. Long-term adherence to low carbohydrate-high protein diets have been associated with an increased risk of developing type 2 diabetes, but total protein and fasting blood glucose do not seem to be associated [61]. The association between dietary protein intake and cardiometabolic health is further complicated as the source of dietary protein seems to be important as well. For example, from a systematic review, it was concluded that there is a reversed association between plant protein and soy protein on blood pressure and LDL cholesterol, respectively [61]. Whereas, diets high in proteins from red meat appear to increase the risk of coronary heart diseases and diets high in plant proteins appear to prevent cardiovascular diseases [66].

#### 3.4.3. Bone Health

The relation between protein intake and bone health has been studied in detail and several meta-analyses have been performed. Pedersen et al. concluded in their systematic review that insufficient evidence is currently available to state an association between protein intake and fractures, bone loss, and osteoporosis [61]. In a more recent systematic review and meta-analysis Wallace at al. concluded that optimal protein intake is key for the development and maintenance of bone throughout the life span and that even protein intake above the recommended daily allowance (RDA) can be beneficial to prevent hip fracture and the loss of bone mineral density [20]. A similar conclusion was formulated after a systematic review and meta-analysis by Shams-White et al. [19]. They observed a positive association between bone mineral density and protein intake, especially in lumbar spine bone mineral density (BMD). 

#### 3.4.4. Cancer

The association between protein intake and cancer risk remains inconclusive. Low protein diets have been associated with lower levels of IGF-1 which could delay aging and cancer [21,67,68]. To interpret these studies, it is of paramount importance to consider the protein source. Indeed, when reported, animal-based protein in diets often worsen the negative association between protein intake and health outcomes, whereas plant-based protein nullified or even reversed these trends [21,61]. For example, Pedersen et al., concluded that there is no effect between overall dietary protein intake and breast cancer, whereas Wu et al., concluded that diets high in dietary proteins from red meat increase the risk for breast cancer and proteins from soy and skim milk reduce the risk of breast cancer [61,69]. Even so, it has to be taken into consideration that a plant-based diet is also associated with other beneficial lifestyle habits including high physical activity and non-smoking, which could potentially confound this association.

Overall, high protein diets can have beneficial effects on cardiometabolic outcomes and bone health, mainly via the positive effect on adiposity. Nevertheless, there also have been concerns about the adverse effects of high dietary protein intake on organ functioning. High protein intake for a long period of time (>2 g/kg/bw) could potentially harm the liver, kidney and gastrointestinal tract [70]. Indeed, high dietary protein intake can lead to glomerular hyperfiltration which can contribute to the progression of kidney disease [71] and protein intake was associated with higher prevalence of non-alcoholic liver disease [72]. Importantly, different protein sources appear to have different health effects and should be taken into consideration.

## 4. Dietary Protein Intake, Physical Activity, and Frailty

### 4.1. Physical Activity and Frailty

There is ample evidence showing that physical activity and exercise supports healthy aging. Adequate physical activity reduces the risk for multiple diseases, such as various cancers, cardiovascular diseases, obesity and type 2 diabetes [73]. In addition, adequate physical activity is associated with reduces risk for falls, disability, activities of daily living (ADL) dependency, institutionalization and hospitalization and, as such, declines the frailty risk at an older age. Unfortunately, frail individuals tend to have low physical activity levels. Using the NHANES data Kehler and Theou demonstrated that frail individuals are less likely to meet physical activity guidelines and show prolonged bouts of sedentary time [74]. An increase in physical activity or exercise training would significantly improve skeletal muscle mass, strength, physical performance, cognitive performance, and psychological wellbeing and has been shown to be a very promising intervention to counteract frailty [75].

Resistance-type exercise training is a very effective intervention to counteract physical frailty as it initiates muscle hypertrophy and improvements in muscle strength and physical performance [76,77,78,79,80,81]. A meta-analysis of 49 randomized intervention studies showed that after an average of 20.5 weeks of resistance-type exercise training, elderly people gained 1.1 kg (CI: 0.9–1.2) of lean body mass [82]. Furthermore, an additional meta-analysis showed that elderly people improved maximal leg press strength by 29 ± 2% and leg extension strength by 33 ± 2% after an average of 18 weeks of resistance-type exercise training [82]. Endurance-type exercise training may also be effective to counteract frailty as it improves mitochondrial biosynthesis, maximal aerobic capacity as well as maximal walking speed in frail older adults [83,84]. The combination of both resistance-type and endurance-type exercise training, however, may be superior to counteract frailty as compared to resistance-type or endurance-type exercise training alone [83,85], although more studies are needed to confirm this. A large study study (*n* = 1634) in this field is the Lifestyle Interventions and Independence for Elders (LIFE) study [86]. This study showed that a combination of resistance, endurance, flexibility and balance exercises is highly effective to counteract physical frailty and prevent mobility disability [86]. But not only physical components of frailty may be improved after exercise, also cognitive performance, mood, emotion, and social networking were improved after multicomponent exercise interventions in frail individuals [87,88]. Based on the available evidence, recommendations for frail older people should include a progressive program of both resistance, endurance, flexibility and balance exercises to prevent and/or counteract both the physical and multiple component frailty phenotype [84].

### 4.2. Protein and Exercise

It has been suggested that exercised muscles become more sensitive to nutrients, allowing more of the available amino acids to be synthesized into skeletal muscle protein. In sedentary, mostly frail elderly subjects, however, the sensitivity of skeletal muscle tissue to anabolic stimuli such as physical activity or protein intake might be reduced [89,90,91]. To illustrate, postprandial rates of muscle protein synthesis were significantly reduced by 26% after a 14-day reduction in physical activity in older adults [89]. To overcome this anabolic resistance, a combination of exercise with increased protein intake seems to be the most plausible strategy. In fact, Pennings et al. showed that resistance exercise after dietary protein provision resulted in a 28% increase in muscle protein synthesis in older adults [92]. In a long-term study, Tieland et al. showed that 24-week protein supplementation (15 g milk protein twice daily provided at breakfast and lunch) combined with resistance training increased muscle mass, strength, and physical performance in frail elderly participants [93]. Furthermore, they were also able to show the benefits of protein and exercise on cognitive performance in frail subjects [94]. These findings also have been shown in multiple meta-analysis, showing an effect of protein supplementation during exercise on muscle mass and strength in older adults [95,96,97]. In contrast, however, Chale et al. could not replicate the beneficial effects of dietary protein supplementation (40 g milk protein per day) during resistance exercise in mobility-limited, frail older adults [98] and also a few systematic reviews were not able to show benefits of protein during exercise [99,100]. A possible explanation for the apparent differences between studies might be the used dietary strategy and the population included in the trials. For example, the amount, timing of protein provision and the source of protein differs between studies and may be of critical importance to find significant results. In addition, those participants who are malnourished may be more susceptible to an increase in protein intake compared to those who have a protein intake well above the recommended daily allowance (RDA > 0.8–1.0 g/kg/d). Also hospitalized subjects, migrants, sarcopenic-obese subjects or elderly in rehabilitation who may have suboptimal nutritional intakes may benefit from additional protein intakes during exercise training. Clearly, more research is warranted in those frail populations allowing us to gain better insight in the potential benefits of additional protein intake during exercise on muscle mass, strength, physical performance and frailty indices, and, as such, support healthy aging, even for the very frail. 

## 5. Discussion

To prevent frailty, research has focused on factors that contribute to the development of frailty. Nutrition and physical activity are factors closely related to frailty. Most efforts so far have been spent on understanding the association between protein intake with or without physical activity and physical aspects of frailty. Far less is known on the association between protein intake and the broader concept of frailty, covering in addition to the physical domain: cognition, mood, social health and comorbidity. We, therefore, performed a narrative review to investigate the association between protein, physical activity and the broader concept of frailty. We identified several observational studies, clinical trials, systematic reviews, and meta-analysis that described the association and effect of protein, with or without physical activity, with/on physical frailty. Most, but not all, find that low protein intake is associated with a higher prevalence and incidence of frailty. Concluding that especially in combination with physical activity optimal dietary protein intake can help to prevent or ameliorate physical frailty. Far less is known on the broader concept of frailty and its association with protein and physical activity. The few studies that do look into this association find a clear beneficial effect of physical activity but no conclusions regarding protein intake can yet be made. Similar, for other important aspects of frailty including mood, cognition, and comorbidity the number of studies are limited and results are inconclusive. 

At this moment there is an ongoing debate on the effect of protein on health domains and the associated recommendations for intake. On one hand, the suggested increase in dietary protein intake for elderly people (up to 1.5 g/kg/bw) may be beneficial for some health outcomes including skeletal muscular health and weight maintenance but on the other hand long term consumption of high protein (2 g/kg/bw) has been associated with adverse effects including renal, digestive, and vascular abnormalities [70]. Future research should focus on including broader measures of frailty and health to better understand the long term beneficial and possible harmful effects of high dietary protein consumption. In addition, more knowledge is required on several aspects. 

First, to optimize dietary protein intake to improve muscle mass, physical functioning, and physical frailty, the amount of protein per meal should be considered. Dietary protein intake in frail elderly at breakfast (on average 10 g), lunch (on average 15 g) or prior to sleep (on average 5 g) may be insufficient to maximally stimulate muscle protein synthesis rates as frail elderly people could be resistant to such low anabolic stimuli [101,102]. Indeed, compelling evidence shows that older adults have lower muscle protein synthesis rates than their younger peers when consuming low (e.g., 10–20 g) amounts of protein per meal [101,102]. This age-related decline in protein synthesis could be explained by low physical activity, blood flow, testosterone or estrogen levels and increased inflammation and/or insulin resistance [103,104]. Therefore, a stronger stimulus, i.e., higher protein (>25–30 g) per meal may be needed to optimize dietary protein synthesis and support muscle growth in frail elderly people [105]. 

Second, the source of protein may be important for frailty. Sources that are rich in essential amino acids (EAA) are suggested to be superior to stimulate muscle protein synthesis in comparison to sources that are lower in EAA [106,107]. Especially, sources that are rich in the essential amino acid leucine, that provides a key signal to initiate muscle protein synthesis. Mainly animal protein sources are rich in EAA and leucine and may, therefore, be the preferred source for frail elderly people [106,107,108]. However, not all studies do observe stronger beneficial effects of animal protein than for plant protein on frailty outcomes [37,109,110]. It could be that proteins from animal food sources are in theory more beneficial for muscle health, but also related to a less optimal dietary pattern (e.g., high in red meat and saturated fats) and positive energy balance. A diet high in plant proteins is generally associated with a more optimal dietary pattern which could beneficially influence frailty. Indeed, several observational studies suggest that a healthy dietary pattern is associated with lower physical frailty prevalence and incidence [36,111,112,113]. Similar, with changes in population and dietary recommendations that have led to a tremendous increase in the demand for protein, mainly from animal sources [114], we have to consider the sustainability of protein consumption. An increase in proteins from animal food sources is associated with increased greenhouse gas emissions and overutilization of water. This further highlights the importance to find sustainable and healthy solutions to optimize protein consumption in elderly people. 

Third, in addition to the amount of protein, also the timing of protein ingestion may be important to optimize dietary protein intake in frail elderly people. Indeed, some intervention studies [115,116] but not all [117,118,119], showed that distributing protein intake evenly throughout the day better stimulates muscle protein synthesis and compared to an unequal protein distributed intake. Especially, long term, well-controlled intervention trials are needed to identify the optimal protein amount, sources and distribution for physically frail older adults. Fourth, we observed that most studies are performed among relatively healthy often Caucasian individuals. Far less is known on for example the optimal dietary protein requirement in frail elderly people with or without the combination of physical activity.

In conclusion, numerous well-performed studies show a beneficial effect of protein intake, especially in combination with physical activity on several aspects of frailty including physical frailty, bone health and risk factors associated with weight loss and maintenance. Nevertheless, the overall effect of protein intake on multidimensional frailty currently not well understood and should be further investigated. Furthermore, research should focus on protein amount, timing/distribution and source to better understand the relation between protein intake, physical activity, and frailty.
